# A microsurgical procedure for middle cerebral artery occlusion by intraluminal monofilament insertion technique in the rat: a special emphasis on the methodology

**DOI:** 10.1186/2040-7378-6-6

**Published:** 2014-06-06

**Authors:** Aslan Güzel, Roland Rölz, Guido Nikkhah, Ulf D Kahlert, Jaroslaw Maciaczyk

**Affiliations:** 1 Department of Neurosurgery, Bahcesehir University, MedicalPark Hospital, 27060 Sehit Kamil, Gaziantep, Turkey; 2 Department of Neurosurgery, University Medical Center Freiburg, Breisacher Strasse 66, 79106 Freiburg, Germany; 3 Department of Stereotactic Neurosurgery, University Medical Center Erlangen, Schwabachanlage 6, 91054 Erlangen, Germany; 4 Department of Neurosurgery, University Medical Center Duesseldorf, Moorenstrasse 5, 40225 Duesseldorf, Germany; 5 Department of Pathology, Division of Neuropathology, Johns Hopkins Hospital, 400 N Wolfe Street, Baltimore 21231, USA

**Keywords:** Middle cerebral artery occlusion, Monofilament, Rat, Surgical anatomy

## Abstract

**Introduction:**

Although there are many experimental studies describing the methodology of the middle cerebral artery occlusion (MCAO) in the literature, only limited data on these distinct anatomical structures and the details of the surgical procedure in a step by step manner. The aim of the present study simply is to examine the surgical anatomy of MCAO model and its modifications in the rat.

**Materials and methods:**

Forty Sprague-Dawley rats were used; 20 during the training phase and 20 for the main study. The monofilament sutures were prepared as described in the literature. All surgical steps of the study were performed under the operating microscope, including insertion of monofilament into middle cerebral artery through the internal carotid artery.

**Results:**

After an extensive training period, we lost two rats in four weeks. The effects of MCAO were confirmed by the evidence of severe motor deficit during the recovery period, and histopathological findings of infarction were proved in all 18 surviving rats.

**Conclusion:**

In this study, a microsurgical guideline of the MCAO model in the rat is provided with the detailed description of all steps of the intraluminal monofilament insertion method with related figures.

## Introduction

Rat models of focal cerebral ischemia are widely used in experimental studies aiming at the elucidation of pathophysiological mechanisms of stroke and the evaluation of new therapeutic approaches in the treatment of occlusive cerebrovascular diseases [1-12].

Among the endovascular techniques of middle cerebral artery occlusion (MCAO), the suture occlusion method is the most frequent experimental paradigm that has been used over the last 20 years [13]. The basis of this procedure consists in the blocking of the blood flow into the MCA with an intraluminal suture (nylon monofilament) inserted through one of the big arteries of the neck, as described before [6,13-16]. If properly performed, this technique provides reproducible MCA territory infarction [4,14,15,17]. It allows transient occlusion with following cerebral reperfusion by retracting of the suture and thereby, different levels of lesion severity depending on the occlusion time can be obtained [12,18-24].

Albeit its common use, getting started with this model in research is difficult. Therefore, we provide here the detailed description of all steps of the modified intraluminal monofilament method with an array of related figures.

## Materials and methods

All surgical procedures were performed in accordance with our institutional guidelines and the German animal protection legislation, under the operating microscope (SMED-Studer Medical, Engineering-AG, Switzerland Yasargil System, VM-900) Female Sprague-Dawley rats (250 to 280 grams) were housed under 12-h light/12-h dark conditions; under temperature of 22-24°C and with food and water ad libitum. The animals were allowed to acclimatize for 2 weeks prior to experiment and were fasted overnight with free access to water, before the surgery.

The animals were anesthetized by 10 mg/kg i.p Ketamine hydrochloride (Ketamine® 10% Essex Pharma GmbH, Germany) and 5 mg/kg i.p Xylazine hydrochloride (Rompun®, Bayer AG, Germany) given intraperitoneally.

The animals were not intubated and blood gases were not monitored during the MCAO.

All procedures were in concordance with German animal law regulations. The animal protocol granted by the Regierungspraesidium Freiburg as well as the ethical commission of the Faculty of Medicine in the University of Freiburg gave ethical permission to perform the described experiments.

### Surgical technique

**Figure 1:**
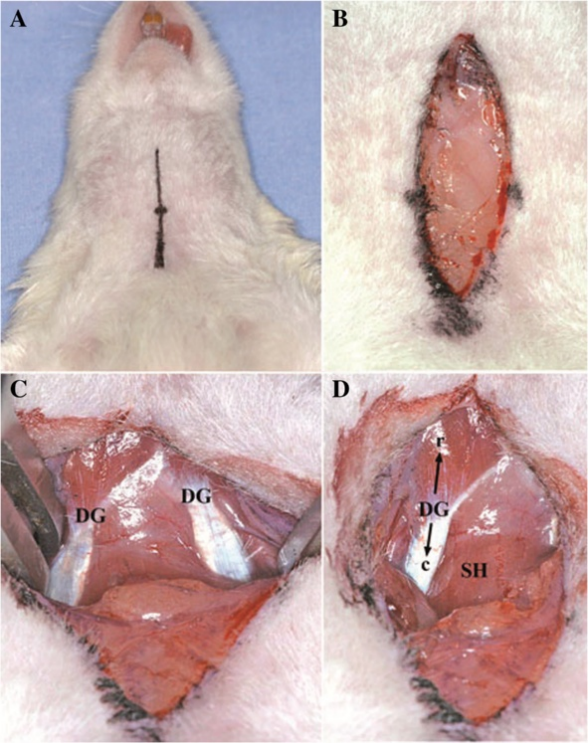
**Location of the skin incision visualized by surgical marker (A).** Dissection of platysma and subcutaneous fatty tissue **(B)**. Preparation of right and left digastric muscles (DG), **(C)**. Preparation of the rostral (r) and caudal (c) part of the right DG and the sternohyoid muscle (SH), **(D)**.

**Figure 2:**
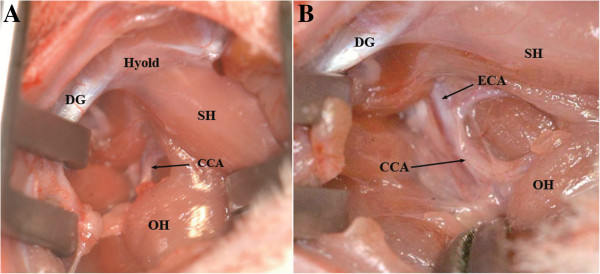
**Retractor between the caudal part of the DG and the Sternomastoid muscle (SM) laterally and the SH medially.** Omohyoid muscle (OH) overlapping the right CCA **(A)**. Caudal mobilization of the OH displays the right CCA **(B)**.

**Figure 3:**
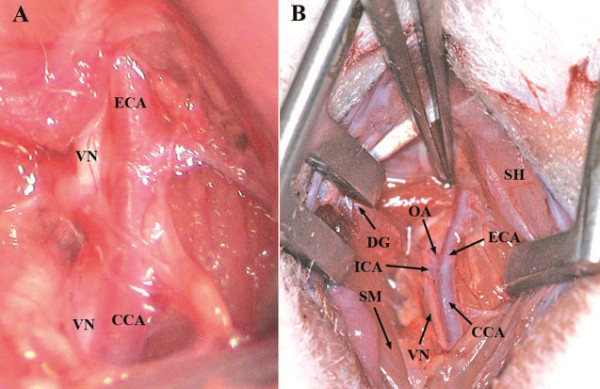
**Identification of anatomical structures: Vagus nerve (VN) located laterally to the CCA (A, B).** occipital artery (OA) originating from ECA very close to the bifurcation of the CCA (B).

We used the following modified surgical procedures which originally were described by several authors [2,6,13,14,16,19,25-27]. Under the operating microscope, a longitudinal cervical midline incision (approximately 2 cm, Figure 1A) through subcutaneous tissue and platysma (Figure 1B) was made. The rostral part of the aponeurosis of digastric muscle (Figure 1C and D) is a good marker allowing the precise localization of the CCA. A self-retaining retractor was positioned between the digastric, sternomastoid and sternohyoid muscles [23,28], (Figure 2A). The omohyoid muscle was gently moved downward to expose the right CCA (Figure 2B). Precise dissection of the perivascular structures (fascia, adventitia and sympathetic plexus) around CCA and ECA (Figure 3A) using the sharp curved forceps was performed. Greatest care was taken in this step to avoid excessive manipulation or lesion of the surrounding neural structures, especially nervus vagus located laterally to the CCA (Figure 3B). In case of bleeding from subcutaneous tissue and surrounding veins, monopolar coagulation has been used.

**Figure 4:**
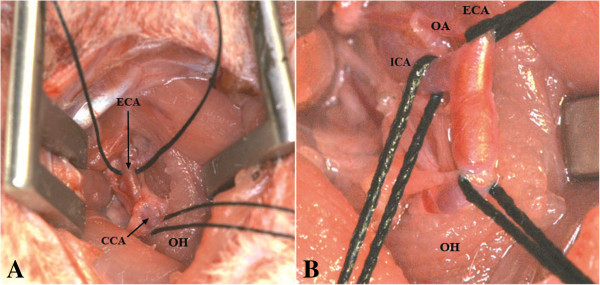
**CCA and ECA hung up by silk suture (A).** ECA and OA hung up together, mobilization of the VN, ICA hung up with silk suture **(B)**.

**Figure 5:**
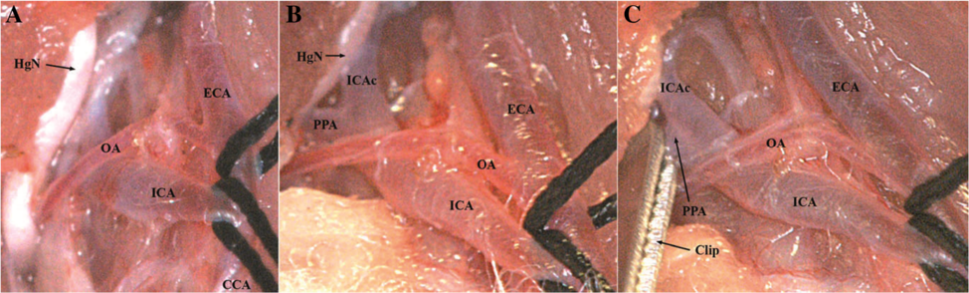
**Preparation of the pterygopalatine artery (PPA), (A).** Dissection of the distal ICA and lateral mobilization of the hypoglossal nerve (HgN), **(B)**. Clip positioned on PPA **(C)**.

The common carotid artery (CCA) was hung up (Figure 4A) with a 6/0 silk suture kept by a haemostatic forceps. The ECA and the OA originating as the first branch of the ECA very close to the bifurcation were hung up together. Then, the ICA was isolated and carefully separated from the lateral adjacent vagus nerve and also hung up by silk suture (Figure 4B). For further dissection of the ICA in the proximity of the skull base, the hyoid bone was carefully lifted with a curved forceps. Here, the PPA, which is the sole extracranial branch of the ICA and its adjacent neural structures, the ansa of the hypoglossal nerve [29], were clearly displayed (Figure 5A,B).

**Figure 6:**
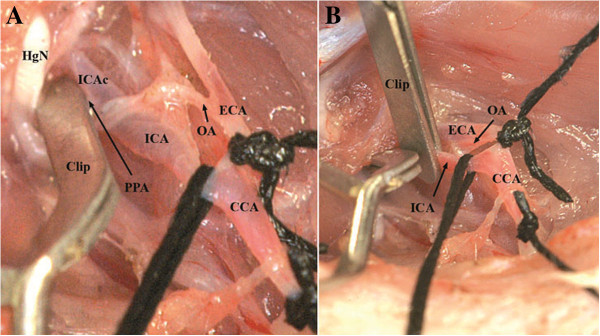
**Ligation of the CCA and ECA/OA (A).** Second clip positioned on the distal part of the ICA **(B)**.

The PPA was clipped with a temporary microvascular clip as described by Kawamura et al. [30], close to its origin from the ICA (Figure 5C). To allow easy introduction of the monofilament into the MCA, the clip was positioned as close as possible to the ICA on the PPA. Afterwards, CCA as well as ECA together with the OA were ligated and subsequently hung up (Figure 6A). A second temporary clip was positioned on the ICA distally of the silk suture (Figure 6B) leaving the biggest distance possible in between. This would later allow pushing the monofilament safely 5–10 mm inside the ICA before removing the temporary clip. Here, it can be transiently fixated by pulling up the nylon suture which provides a crucial advantage as it prevents dislocation of the filament by retrograde blood flow. Blood loss can be reduced or even entirely prevented by this method.

**Figure 7:**
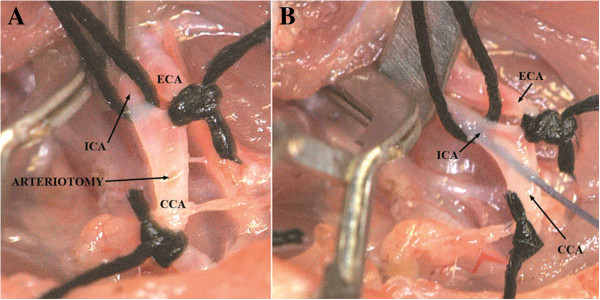
**CCA-arteriotomy below the bifurcation (A).** Monofilament insertion **(B)**.

**Figure 8:**
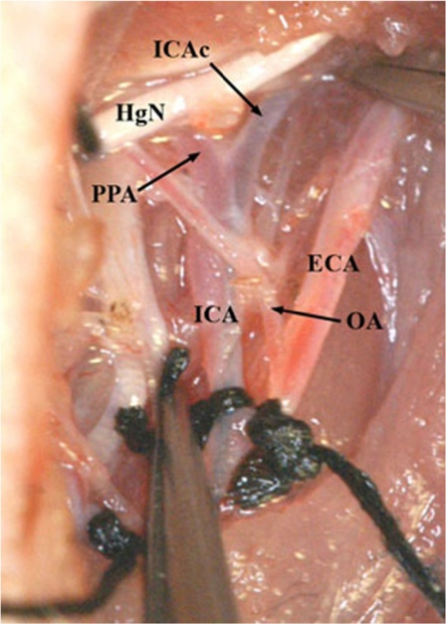
**Monofilament advanced beyond the PPA into the cranial part of the ICA (ICAc) leading to the skull base and ICA ligation.** Clips on PPA and ICA removed.

Now, a small incision (arteriotomy) was made by microsurgical scissors on the CCA approximately 3 mm proximal of the carotid bifurcation [31] (Figure 7A), and the poly-L-ornithine coated filament was inserted into the ICA through the CCA (Figure 7B). Next, the clip on the ICA was removed and the filament was carefully further advanced for approximately 16 to18 mm until mild resistance was felt [2,6,26,32-34], indicating that the tip was lodged in the anterior cerebral artery and thus blood flow to the MCA was blocked, as reported previously [14,19,27,33]. Afterwards, the occluded ICA (with the intraluminal monofilament) was ligated distal to the CCA bifurcation with the 6/0 silk suture (Figure 8). After careful haemostasis the skin incision was closed, leaving 2 cm of the nylon filament protruding. The whole procedure took 20 to 30 minutes for each rat. Animals were allowed to wake up, and clinical evaluation of the lesion was performed. Recovery of consciousness occurred within 30–60 minutes after the operation in all animals.

For temporary MCAO, reperfusion was obtained by withdrawing the suture approximately 13–15 mm after the ischemia time chosen for the experiment until resistance was felt when the tip reached the ligation of the ICA. In the present study, we chose an occlusion time of 60 minutes. The method allows reperfusion of the two distal branches of the ICA; the anterior choroidal and hypothalamic arteries [31,35,36], preventing the possible loss of the experimental animal, as the hypothalamic artery occlusion contributes to hyperthermia after intraluminal suture occlusion which is related to more pronounced ischemic damage and postoperative mortality [36].

### Intraluminal suture preparation

**Figure 9:**
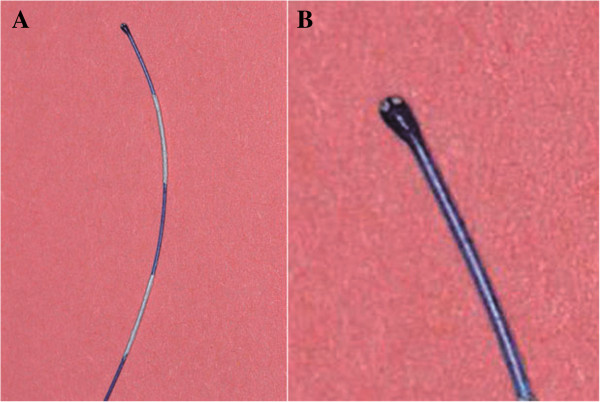
**Poly ornithine coated monofilament marked with white permanent marker in 5 mm distances (A).** Rounded and size-standardized tip **(B)**.

The suture was prepared from a 5 cm-long part of a sterile 4/0 nylon monofilament (Ethilon Nylon Suture, Ethicon Inc. Germany). One end of the suture was rounded carefully by melting with a portable electrocautery unit (Harvard apparatus Ltd, Germany). The end of the suture was therefore kept inside the electrocautery ring for several seconds. Tip diameter was standardized to 0,38-0,40 mm using a micro forge (Narishige MF 900, Japan). To obtain intraoperative control on the length of the intraarterially introduced monofilament, we have marked the proximal 20 mm of the suture with sterile permanent marker in 5 mm distances (Figure 9A,B). To increase the adhesive properties of the nylon suture, it was coated with Poly-L–Ornithine (PLO, Sigma Aldrich, Germany) by immersing in 1%-PLO solution overnight at room temperature as described previously [1].

### Surgical tools

Disposable scalpel No. 10 (Feather company, Japan), 4/0 nylon suture and 6/0 silk suture (Ethicon Inc. Deutschland), Wullstein retractors (No. 17018–11), adson forceps (No. 91106–12), MORIA forceps (Straight, No.11370-40) MORIA forceps (Curved, No. 11370-42), Micro-Mosquito (Straight, serrated, No. 13010-12), Hartman Hemostatic forceps (No. 13002–10), Student iris scissors, Straight No. 91460–11), MORIA spring scissors (Straight, No. 15396–00), 2 micro clips (curved serrefines No. 18055–01 and straight serrefines No.18055-05) Micro-clip applicator (No. 18056-14), Michel suture clips (No. 12040-02), Applying forceps for Michel suture clips (No. 12018-12), Ear punch for animal identification (No. 24210–02) were from FST (Fine Science Tools GmbH, Germany) catalogue.

The illustrations were acquired with the digital camera D2Xs (Nikon, Japan) equipped with the objective NIKKOR AF-S 300 mm f/2,8G ED VR II (Nikon, Japan).

## Results

### Learning experience and potential pitfalls

In a preliminary experiment, 20 rats have been operated and 15 of them died either during the operation or within the first 24 hours (mortality rate of 67.5%). Ten rats died due to intracerebral hemorrhage as revealed by the post-mortem examination of these animals caused probably by the perforation of the ACA beyond the ostium of the right MCA during insertion of the monofilament [6,21]. Three rats died due to bleeding from the big vessels of the neck during early stages of the operation, and two died because of cervical haematoma or haemorrhage leading to compression of the trachea, vascular and neural structures. After extensive training in the separate group (n = 20) we lost only two rats (surgical success rate was 90% (n = 18), and mortality rate was 10%). One animal died due to intracerebral haemorrhage (complication of monofilament insertion), and the other due to ICA bleeding, while we introduced the monofilament through the CCA. 18 of 20 rats survived at least four weeks. All of the surgeries in this study were performed by trained neurosurgeons with extensive micro-surgical experience and a neurosurgery-resident in the second year of the training. Nevertheless, the relative small experience with the rat extracranial vascular anatomy and intraluminal placement of the filament resulted initially in the high rate of perioperative mortality. The length of the necessary training depends clearly on the surgical experience of the investigator. Previous micro-surgical skills unequivocally facilitate a fast development of the MCAO model. In our hands the crucial modification contributing to the safe and reliable occlusion of the MCA and reproducible stroke induction within its perfusion territory was the temporary closure of the PAA preventing an erroneous insertion of the filament into the extracranial ICA branches. Apparently this maneuver has also been applied by researches introducing intraarterial catheters for experimental, intracerebral drug/cells delivery (personal communication P. Walczak/M. Janowski, Johns Hopkins University, Baltimore, USA).

**Figure 10:**
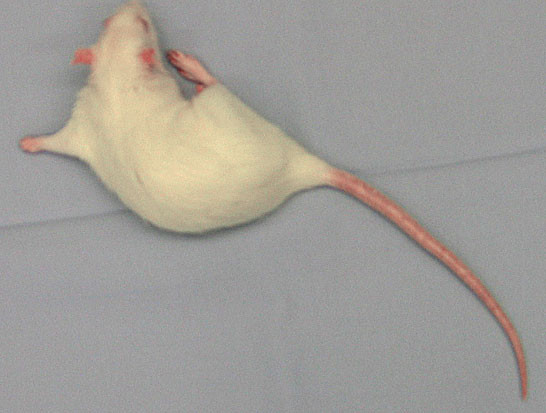
During the recovery period, all surviving rats showed contralateral forelimb paralysis following temporary brain ischemia.

The effects of MCAO were confirmed by the evidence of motor neurological deficit (four points according to the applied scale as shown in Table 1, Figure 10) and histopathological findings of infarction (not shown) in all surviving rats. Interestingly, during the initial training period we could observe a clear dependence of the area of infarction on the time of the MCA occlusion. When the filament was kept in the lumen of the MCA for of up to 30 minutes it resulted frequently in an isolated insult within subcortical structures (CPU) whereas longer closure of the vessel produced larger infarct areas. After an hour of occlusion we could constantly observe a complete cortico-subcortical localization of the stroke. Although not performed in our study, also body temperature monitoring plays a crucial role concerning the reproducibility of the size of the infarcted brain tissue. Hypothermia exerts a neuroprotective influence and therefore can adversely affect the MCAO model leading to diminished magnitude of tissue infarction [37]. Therefore, assuring the normothermic perioperative conditions applying i.e., a feedback controlled heating pad, which warms according to the rectal temperature of the animal, is highly recommended.

**Table 1:** **Neurological evaluation of rats after MCAO**[29]

**Score**	**Evaluation**
0	No apparent deficit
1	Contralateral forelimb flexion
2	Decreased grip of the contralateral forelimb while tail pulled
3	Spontaneous movement in all directions; contralateral circling only if pulled by tail
4	Spontaneous contralateral circling

### Functional outcome

The neurological examination was carried out after full recovery from anesthesia. The rats were assessed for contralateral motor deficit to confirm ischemia by using a previously described scoring method (Table 1) [20]. In the present study, all surviving animals showed clear neurological motor deficits within the first two hours after MCAO (100% percent with score 4).

During the recovery period, all surviving rats showed also forelimb flexion and contralateral forelimb paralysis, confirming the permanent damage following temporary brain ischemia [18].

## Discussion

To produce focal ischemia, the occlusion of the MCA has been the target of most investigations, because this vessel is the most commonly affected in stroke victims [38]. This model has been first introduced by Koizumi et al. [13], and later modified by Longa et al. [14]. Numerous further modifications of this method have been reported in the literature [2,14,15,19,21,25-27]; however, the literature describing the important microsurgical hallmarks of the MCAO and identifying the critical steps and highlighting the possible pitfalls of the surgical technique is very scarce. For MCAO, the filament may be inserted through the ECA, ICA or CCA [6,8,14,21,24,26,27,32,39,40]. Alternatively to the method we applied, MCAO is frequently produced by insertion of the monofilament through the ICA to the origin of the MCA via the ECA. This technique requires coagulation or ligation of the OA [36]. Another significant difference between our operation technique and those described by many other authors is the thorough closure of the PPA that represents a substantial step in our operation protocol. It guarantees the insertion of the monofilament fiber directly into the MCA.

To insert the monofilament through ECA, further dissection of the ECA and its branches is required. Inserting the monofilament through CCA, we minimized the dissection of the ECA and its branches in the area of the carotid bifurcation. Moreover, ligation of CCA facilitates introduction of the monofilament and reduces active hemorrhage and hematoma formation during and after the procedure. The disadvantage of CCA ligation is to provide cerebral blood flow through anterior communication artery instead of ICA.

Along with many advantages like its simple technique, the minimal invasive nature of the procedure, low mortality and redundancy of a craniotomy [41], all the intraluminal suture models of MCAO share the same disadvantages: insertion of the suture occludes the entire course of the ICA, leading to obstruction of the hypothalamic artery (HA). This causes hypothalamic infarction with associated pathologic hyperthermia that confounds the results of the investigation, for instance neuroprotective drug evaluation [6,38,42]. Finally, the anterior choroidal artery can be occluded by the filament, while the lumen of the MCA still allows perfusion. This may cause clinical stroke signs mimicking MCAO. Some other unwanted side effects of this method are subarachnoid hemorrhage, intraluminal thrombus formation, and premature reperfusion [6,21,30]. When carefully carried out, sharp dissection allows a fast and easy approach to the vessels securing their protection at the same time. We suggest reducing the interventions on the vessels and their surrounding structures to a minimum, especially manipulations on the ECA and its branches. As a technique to reduce tissue damage, we strongly recommend the use of a temporary microvascular clip for the occlusion of the PPA as described before [43] instead of a ligation.

Ischemic stroke is a very heterogeneous disorder. In this respect, mimicking all aspects of human stroke in one animal model is not possible. Although ischemic stroke was shown clinically and histologically in this study, volume of infarcted tissue was not measured. If volume of infarcted area could be measured on histologic sections or MRI, the results might be more objective. However, the study was focused on describing the methodology of surgical MCAO, and volume measurement was not planned.

Another limitation of the study is that, some physiological data, such as blood gases and body temperature of animals, were not measured during MCAO experiment.

Intraoperative Doppler ultrasonography could be useful for measurement of cerebral blood flow, however Doppler ultrasonography was not available during the procedure unfortunately.

It is well known that there is a learning curve for a MCAO model. Therefore, before the study, 20 rats were used for training and detailed description of surgical technique. We focused on the occlusion technique and evaluated the results of MCAO on clinical and histological findings. We believe that if all steps of this method is applied correctly, the procedure is sufficient for MCAO in rats.

In conclusion, we present a modified surgical technique for intraluminal MCAO. In comparison to methods described by other authors, our procedure avoids the dividing of the omohyoid muscle. We showed that a gentle dissection and efficient distraction is sufficient to reach the relevant anatomical structures. Furthermore, we reduced the dissection of the ECA and its branches to a minimum in the area of the carotid bifurcation. In our procedure, we did not coagulate the OA but ligated it together with the ECA as this saves time and reduces tissue damage. As mentioned above, we found the microvascular clip to be an excellent way to close the PPA greatly facilitating the introduction of the filament if positioned correctly. We recommend placing it on the PPA as close as possible to the origin of this vessel from the ICA.

## Conclusion

The presented study demonstrates that the microsurgical filament occlusion of the MCA can be easily performed in rats by the above described procedure following some intensive microsurgical training. This modified surgical approach is simple and can be followed easily by the microsurgical guidelines and landmarks provided here. This may promote experimental approaches in stroke that may ultimately advance the scientific progress in experimental, and potentially, also clinical forms of cerebrovascular diseases.

## Competing interests

The authors declare that they have no competing interests.

## Authors' contribution

AG, RR and JM performed the experiments. UDK, JM and GN wrote manuscript and performed discussion in the current scientific context. AG, UDK and JM generated high quality images. All authors read and approved the final manuscript.
